
JOURNAL INFORMATION
==============================
NLM Title Abbreviation: Wirtschaftsdienst
Journal ID: Wirtschaftsdienst
NLM Journal ID: 101086612

Wirtschaftsdienst (Hamburg, Germany : 1949)

ISSN: 0043-6275

ARTICLE INFORMATION
==============================
PMCID: PMC8383021
PMID: 34456383
DOI: 10.1007/s10273-021-2975-5
Article ID: 2975
Article version: 1
Subjects: Zeitgespräch

COVID-19-Pandemie — ethische und ökonomische Herausforderungen für ein gelingendes Krisenmanagement

Biller-Andorno Nikola biller-andorno@ibme.uzh.ch
1
Kapitza Thomas tk@thomaskapitza.de
2
1 grid.7400.3 0000 0004 1937 0650 Institut für Biomedizinische Ethik und Medizingeschichte (IBME), Universität Zürich, Winterthurerstrasse 30, CH-8006 Zürich, Schweiz
2 Sachverständigenbüro Kapitza, Ludwigstraße 3, 82110 Germering, Deutschland
Electronic publication date: 2021 Aug 24
Print publication date: 2021
Volume: 101
Issue: 8
First page: 586
Last page: 590
Copyright: © Der/die Autor:in(nen) 2021
License: Open Access: Dieser Artikel wird unter der Creative Commons Namensnennung 4.0 International Lizenz veröffentlicht (https://creativecommons.org/licenses/by/4.0/deed.de).
Open Access wird durch die ZBW — Leibniz-Informationszentrum Wirtschaft gefördert.
License URL: https://creativecommons.org/licenses/by/4.0/

issue-copyright-statement: © The Author(s) 2021

==============================
Prof. Dr. med. Dr. phil. Nikola Biller-Andorno ist Direktorin des Instituts für Biomedizinische Ethik und Medizingeschichte (IBME) der Universität Zürich und Mitglied der Arbeitsgruppe „Zukunft der Medizin“ der BBAW.

## Die Ziele bestimmen den Weg

In den zurückliegenden 18 Monaten der COVID-19-Pandemie wurde die Komplexität nationalen und internationalen Krisenmanagements deutlich sichtbar. Neben die epidemiologische Herausforderung einer wirksamen Eindämmung der Pandemie trat die Frage der Bewertung verfügbarer Handlungsoptionen in ethischer, ökonomischer und gesellschaftspolitischer Hinsicht. Auch wenn die wachsende Zahl von Menschen mit Impfschutz insgesamt Anlass zur Hoffnung auf eine Entspannung der Krisensituation gibt, bleibt die Situation – insbesondere aus globaler Perspektive – mit Blick auf fairen Zugang zu knappen Ressourcen, die längerfristigen ökonomischen Folgen und den Schutz der Grundrechte prekär.

Auch wenn die Zeit für eine umfassende Retrospektive noch nicht gekommen ist, scheint es vor diesem Hintergrund sinnvoll, bereits jetzt medizinethische und ökonomische Aspekte eines wirksamen und nachhaltigen Pandemiemanagements (PM) näher zu betrachten. Überlegungen zu Management und Governance setzen eine Definition des zu erreichenden Ziels voraus. Dieses soll hier so verstanden werden, dass der gesundheitliche, ökonomische und psychologische Schutzbedarf der Bevölkerung effizient und in ethisch angemessener Weise durch die beteiligten Institutionen abgedeckt wird.

**Prof. Dr. med. Dr. phil. Nikola Biller-Andorno** ist Direktorin des Instituts für Biomedizinische Ethik und Medizingeschichte (IBME) der Universität Zürich und Mitglied der Arbeitsgruppe „Zukunft der Medizin“ der BBAW.

Die zurückliegenden Monate haben die überragende Bedeutung eines staatlich aufgebauten und gesteuerten Pandemiemanagements zur Bewältigung der COVID-19-Gesundheitskrise wie auch die Bedeutung von privatwirtschaftlichen Initiativen und Public-Private-Partnerships aufgezeigt. Es wurde deutlich, dass das aktuelle Pandemiemanagement vor allem vier Themenfelder berücksichtigt:

- Ermöglichung und Förderung von Forschung über die medizinische Bekämpfung der Virusvarianten zur Bereitstellung und Verteilung eines zuverlässigen Infektionsschutzes und zur wirksamen klinischen Versorgung akut erkrankter infizierter Menschen und ihrer Nachversorgung (gesundheitliche Daseinsvorsorge unter Pandemiebedingungen).
- Sicherstellung der prognostisch benötigten medizinisch-pflegerischen Versorgungskapazitäten bei den Versorgungseinrichtungen und Leistungserbringern im Gesundheitssektor (Sicherstellung der Versorgungsbereitschaft und der Versorgungskapazitäten).
- Bereitstellung und Verteilung finanzieller Mittel, finanzieller Entlastungen und von Dienstleistungen zur Bewältigung der wirtschaftlichen Auswirkungen der Pandemie (Stabilisierung der Volkswirtschaft und Schutz der betroffenen wirtschaftlichen Interessengruppen).
- Ermöglichung eines gesamtgesellschaftlichen Pandemiediskurses zur Information und Begleitung der Bevölkerung in den persönlich einschränkenden und belastenden Krisenzeiten.

Die Herausforderungen für das Pandemiemanagement und inhaltliche Ansatzpunkte für PM-Ziele sind in Tabelle 1 übersichtsmäßig dargestellt.

Pandemien entfalten eine alle gesellschaftlichen Bereiche und Wirtschaftssektoren beeinträchtigende Wirkung. Pandemien sind damit, zumindest temporär, sowohl Gesund-

Tabelle 1
**Herausforderungen und Zieloptionen des Pandemiemanagements (PM)**

| PM-Stufe | Herausforderungen | Ansatzpunkte für PM-Ziele (Auswahl) |
|---|---|---|
| I<br>Schaffung der Ressourcenvoraus-setzungen | Mittelaufbringung | • Staatliche (reserven-, schulden- und entlastungsbasierte Zwischen-)Finanzierung des gesamtgesellschaftlichen Pandemieaufwands<br>• Mittelzuweisungen für pandemierelevante Forschung usw.<br>• Beiträge wirtschaftlich leistungsfähiger Bevölkerungsgruppen (Solidarfinanzierungen) |
| II<br>Festlegung und Umsetzung der Lösungsansätze | Mittelallokation: primäre (medizinische) Pandemie-bekämpfung | • Medizinisch-pflegerische Akutversorgung erkrankter/infizierter/gefährdeter Menschen<br>• Infektionsschutz für Bevölkerung (global) |
| | Pandemiebekämpfung durch sektorale Versorgungsbereit-schaft | • Sicherstellung der Versorgungsbereitschaft (Personal, Sachmittel, Liquidität, Versorgungskompetenz der nationalen Gesundheitsinfrastruktur)<br>• Sicherstellung der prognostizierten Versorgungskapazitäten (medizinisch-pflegerische Infrastruktur, Patientenlogistik, katastrophenmedizinische Kapazitäten) |
| | Bekämpfung kollateraler Pandemieeffekte | • Vermeidung eines Zusammenbruchs des Finanzsystems (Erhalt Liquidität und Zahlungsfähigkeit) der Akteure einschl. privater Haushalte<br>• Verfügbarkeit lebenswichtiger Ressourcen (Lebensmittel, Wasser, Energie)<br>• Schutz der ökonomischen Wertschöpfungs-, Produktions- und Lieferketten |
| | Pandemiebekämpfung durch Systemstabilisierung | • Stabilitätserhalt des politischen Systems einer freiheitlichen Demokratie<br>• Erhalt der öffentlichen (einschließlich militärischer) Sicherheit<br>• Weiterentwicklung technologischer Fähigkeiten (BioTech, Pharma, Digitalmedizin)<br>• Bewahrung verfassungsrechtlicher Freiheitsrechte<br>• Schutz des Wohlstands-, Wohlfahrts- und Sozialniveaus<br>• Bewältigung pandemiebedingter Emigration/Immigration und Vermeidung gesellschaftlicher Konfliktpotenziale (Eingliederung, Verdrängung, Bekämpfung) |

Quelle: eigene Darstellung.

heitskrisen als auch Wirtschaftskrisen. Deshalb ist ein nationales wie internationales Pandemiemanagement notwendig, das über den Gesundheitssektor hinaus die Sicherheit der Bevölkerung und die Stabilität des Wirtschaftssystems im Blick hat (Kapitza, Greiff und Mann, 2020).

Sowohl im Gesundheitssektor wie in der nationalen und globalen Ökonomie ist wirksames Pandemiemanagement darauf angewiesen, die Sichtweisen, Einschätzungen, Interessen und Entscheidungen der Beteiligten untereinander abzustimmen. Durch transparente Information, Teilhabe und Kooperation können Vertrauen und Solidarität und damit die Resilienz von Individuen, sozialen Gruppen und Bevölkerungen gestärkt werden. Die Bereitschaft der Bevölkerung, ein wirksames Pandemiemanagement mit seinen einschränkenden und Verhaltensveränderungen erfordernden Vorgaben zu akzeptieren, ist davon abhängig, ob es die moralischen Auffassungen und Werte der Betroffenen berücksichtigt und vertrauenswürdig erscheint.

## Ethisches Pandemiemanagement: Akzeptanz als Wirksamkeitschance

Die Akzeptanz von Managementansätzen zur COVID-19-Pandemie, insbesondere bezüglich der Steuerungsmaßnahmen und der Entscheidungstragenden, beruht auch auf einer positiven wertebezogenen Einschätzung der Betroffenen der festgelegten Ziele und kommunizierten Prioritäten des Krisenmanagements (Biller-Andorno, 2020; Spitale et al., 2020). In Tabelle 2 werden diese Aspekte in Form einer Übersicht dargestellt.

Wie die Übersicht zeigt, gibt es eine Vielzahl ethischer Werte, die auf den unterschiedlichen Zielebenen (Individuum, Gesundheitssektor, Volkswirtschaft, Nationalstaat bzw. supranational) des Pandemiemanagements von Bedeutung sein können. Wirksames Pandemiemanagement sollte sich nicht nur auf eine ethisch adäquate Allokation von materiellen Ressourcen fokussieren. Vielmehr sollte – auf Vertrauenswürdigkeit basierendes – Vertrauen in seine Absichten und seine Leistungsfähigkeit angestrebt werden, damit dieses Vertrauen als eine immaterielle Basisressource für ein wirksames nationales und internationales Pandemiemanagement zur Verfügung stehen kann. Diese Ermöglichung begründeten Vertrauens kann ein wichtiges Merkmal einer Good Governance des Pandemiemanagements sein. Ein in ethischer Hinsicht qualitativ hochstehendes Pandemiemanagement sollte deshalb folgende Aspekte berücksichtigen:

- Ist den Pandemiemanagement-Verantwortlichen bewusst, dass ihre moralische Integrität die Effektivität und Effizienz der Pandemiemaßnahmen stärken kann

Tabelle 2

**Zieloptionen und Werte des Pandemiemanagements (PM)**

| Mögliche PM-Ziele | Ethische Werte | Mögliche Wirkungen |
|---|---|---|
| Mittelgenerierung<br>• Finanzierungsziel (Pandemieaufwand)<br>• Finanzierung pandemierelevante Forschung usw.<br>• Aufbau von Solidarfinanzierungen | Legitimität<br>Glaubwürdigkeit<br>Adressierbarkeit Verantwortung<br>Transparenz | Durchsetzbarkeit<br>Akzeptanz<br>Nachvollziehbarkeit |
| Medizinische Pandemiebekämpfung<br>• Gesundheitliche Daseinsvorsorge (unter Pandemiebedingungen) | Fair Process (Priorisierung, Triagierung) | Leidensverminderung<br>Nachvollziehbarkeit |
| Sektorale Versorgungsbereitschaft<br>• Sicherstellungsziel (Versorgungsbereitschaft und Kapazitätsbedarf) | Zugangsgerechtigkeit | Versorgungssicherheit |
| Kollaterale wirtschaftliche Pandemieeffekte<br>• Wirtschaftliche Daseinsvorsorge (Volkswirtschaft)<br>• Versorgungssicherheit (Lebensmittel, Wasser, Energie)<br>• Prozesssicherheit (Wertschöpfungs-, Produktions- und Lieferketten) | Existenzschutz<br>Fairness (bedarfsadjustierte Hilfe)<br>Transparenz<br>Erklärbarkeit<br>Adressierbarkeit Verantwortung | Akzeptanz<br>Nachvollziehbarkeit<br>Partizipation<br>Kontrollierbarkeit<br>Widerspruchsoption |
| Systemstabilisierung<br>• Systemstabilität (Demokratie + Freiheitsrechte)<br>• Öffentliche (einschließlich militärischer) Sicherheit<br>• Fortschrittsfähigkeit (BioTech, Pharma, Digitalmedizin)<br>• Stabilität des Wirtschafts- und Sozialsystems<br>• Gesellschaftliche Konfliktpotenzialminimierung | Verteilungsgerechtigkeit<br>Fairness<br>Transparenz<br>Sinnhaftigkeit<br>Empathie<br>Erklärbarkeit<br>Partizipation | Akzeptanz<br>Tolerierung<br>Nachvollziehbarkeit<br>Partizipation<br>Kontrollierbarkeit<br>Widerspruchsoption |

Quelle: eigene Darstellung.

und damit eine Generierung von persönlichem Vertrauen in die Beteiligten und die öffentlichen Institutionen ermöglicht wird?

- Welche normative Zielstruktur, Zielhierarchie bzw. Zielprioritäten und einzelnen Werte werden für das Pandemiemanagement festgelegt und im weiteren Verlauf gegebenenfalls modifiziert? Sind die normativen Festlegungen plausibel und nachvollziehbar begründet?
- Gibt es definierte Ergebniskategorien, die sowohl immaterielle Zielgrößen (z. B. Leidensminimierung oder Ermöglichung individuell als sinnvoll empfundener Lebenschancen) als auch ökonomische Zielgrößen (z. B. einzusetzende finanzielle Ressourcen für Einzelmaßnahmen der Pandemiebekämpfung) berücksichtigen?
- Sind die getroffenen Entscheidungen ethisch und inhaltlich-rational begründbar und erklärbar, und sind die auf ihnen beruhenden konkreten Maßnahmen transparent für alle Beteiligten und Betroffenen? Werden mögliche Interessenkonflikte bei Entscheider:innen (insbesondere Politik) und Entscheidungsvorbereitenden (insbesondere Wissenschaft und Lobbygruppen) berücksichtigt?
- Sind die PM-Maßnahmen für die gesamte Bevölkerung verbindlich, und können damit politische, gesellschaftliche, wirtschaftliche und medizinethische Free-Rider-Effekte eingegrenzt werden, welche die Pandemie als Vorwand zur Durchsetzung gruppen- oder eigennütziger Interessen nutzen?
- Wird das Pandemiemanagement regelmäßig evaluiert sowie hinterfragt und dabei die Schließung der epistemischen Lücke bezüglich steuerungsrelevanter Pandemiedaten angestrebt (z. B. Massendaten, Indikatoren, Key Performance Indicator (KPI) als Frühwarn-, Alert- und Monitoring-Grundlagen)?

## Medizinethischer Bezugsrahmen für Allokationsprozesse

Die Sicherstellung eines fairen Zugangs zu knappen klinischen Behandlungsmöglichkeiten oder zu eingeschränkt verfügbaren Impfstoffen sind typische Fragestellungen im Pandemiemanagement. Hier bringt die Medizinethik ihre Lösungskonzepte und wissenschaftlichen Überlegungen ein. Allokationsentscheidungen im Kontext der Pandemie betreffen insbesondere Entscheidungen über die Zuteilung von (überlebens-)wichtigen Behandlungsmöglichkeiten und Impfstoffen zu jeweiligen Bedarfsgruppen in den jeweiligen Bevölkerungen. Materielle Kriterien zur konfliktminimierenden Regelung (Badano, 2018) dieser Zuteilung von Versorgungs-, Heilungs- und Lebenschancen sollten im Pandemiemanagement genutzt werden. Das Konzept der Accountability for Reasonableness (A4R) schlägt eine Kriterienliste für die Angemessenheit eines Allokationsprozesses vor, welche sich im Zusammenhang mit globalen Verteilungsfragen im Gesundheitsbereich bereits vielfach bewährt hat (Daniels, 2008):

- *Publicity Condition (Transparency)*, hier verstanden als Öffentlichkeitsbedingung zur Transparenzsicherung;
- *Relevance Condition (Stakeholder Involvement)*, hier verstanden als Bedeutungsbedingung für die Einbeziehung der Anspruchsgruppen;
- *Revision and Appeals Condition*; hier verstanden als Überarbeitungs- und Einspruchsbedingung;
- *Regulative Condition (Enforcement)*, hier verstanden als hoheitliche, reguliererbezogene Durchsetzung der getroffenen Regelungsentscheidungen;

Die vier Kriterien sollen einen fairen Allokationsprozess im Gesundheitssektor im Rahmen des Pandemiemanagements ermöglichen. Prozedurale Fairness wird damit zum Erfolgsfaktor. Hintergrundannahme ist dabei, dass in Anknüpfung an Rawls' Konzept der Fair Equality of Opportunity (Rawls, 1999) die Entscheidungen im Gesundheitswesen so getroffen werden sollen, dass alle die gleiche Chance haben, ihre Lebenspläne zu verwirklichen. Allerdings ist diese Vorgabe zu abstrakt, um als Grundlage dafür zu dienen, welche substanziellen normativen Kriterien bei Entscheidungen im Einzelfall zum Zuge kommen sollen. Daher kommt der prozeduralen Komponente im Fall von Allokationsentscheidungen besondere Bedeutung zu. Die Berücksichtigung der A4R-Bedingungen ermöglicht Entscheidungstragenden, Allokationsentscheidungen zumindest zum Teil mit Verweis auf den fairen Prozess ihrer Entstehung zu legitimieren.

Ein Merkmal ethischen Pandemiemanagements ist es somit, dass die knappheitsbedingt notwendigen Allokationsprozesse nicht eine vertrauensstörende Umsetzung von Klientel- und Gesellschaftspolitik mit anderen Mitteln sind. In einer gesamtgesellschaftlichen Krisensituation, in der rationale Entscheidungen zur materiellen Ressourcenzuteilung organisiert und getroffen werden müssen, ist die Qualität und Akzeptanz hoheitlicher Entscheidungen abhängig von der ethisch-normativen Qualität der zugrunde liegenden Entscheidungsprozesse.

Medizinethische Überlegungen können daher nicht als komplexitätserhöhende Nice-to-Have-Überlegungen gedanklich vernachlässigt werden. Vielmehr sind sie die unverzichtbare Grundlage intrinsischer Akzeptanzbereitschaft bei Beteiligten und Betroffenen hinsichtlich der jeweiligen Allokationsentscheidungen bzw. den mit ihnen verbundenen Konsequenzen. Werteüberlegungen werden damit zum immateriellen und unsichtbaren Erfolgsfaktor für die Durchführung und Umsetzung wichtiger Entscheidungen des Pandemiemanagements.

## Ausblick

Die Bewältigung der COVID-19-Pandemie ist eine Herausforderung, die nur durch intensive Zusammenarbeit über Sektoren und Nationen hinweg gelingen kann. Medizinethische Überlegungen stellen die Grundlage für eine gute Governance, verstanden als moralisch begründetes, zielorientiertes, transparent gerechtfertigtes Handeln, dar. Insbesondere für die heiklen Allokationsentscheidungen bieten medizinethische Konzepte und Grundlagen eine nachvollziehbare, gemeinsame Rahmensetzung für alle im Allokationsprozess Beteiligten an. Auf diese Weise kann das Spannungsverhältnis zwischen den Schlüsselprinzipien Nutzenmaximierung und Gerechtigkeit (Joebges und Biller-Andorno, 2020) durch prozedurale Kriterien eines fairen Prozesses bewältigt werden.

Eine Pandemie kann durch nationales Handeln allein nicht bewältigt werden. Die Bearbeitung der Themen- und Aktionsfelder eines supranationalen Pandemiemanagements kann weiter verbessert werden,

- wenn die globale Gesundheitsgovernance gestärkt wird (z.B. die Zulassung der Impfstoffe erst über die WHO, dann national),
- wenn Verantwortlichkeiten für Allokationsentscheidungen durch transparentes Abwägen relevanter Argumente, Einspruchsmöglichkeiten und konsequente Umsetzung (A4R) adressiert werden,
- wenn die Erwartungen an privatwirtschaftliche Akteure bzw. zuliefernde Industrie definiert (z.B. COVAX als privilegierter Partner (Berkley, 2020)) und Anreize gestaltet werden,
- wenn die Staatengemeinschaft ihren gemeinsamen globalen Beitrag als Leistung (*health diplomacy*) würdigen (Vermeidung von Impfrennen und Exportrestriktionen) und nationale Interessen eingehegt werden (z.B. Caps für bilaterale Advance Purchase Agreements),
- wenn die Zustimmung der Bevölkerung zu den getroffenen Entscheidungen im globalen Pandemiemanagement (*citizen commitment*) durch Kommunikation und Partizipation gefördert wird.

Es gilt, gemeinsames Handeln in der Gesundheitskrise und darüber hinaus zu unterstützen. Alle Beteiligten können sich die Konsequenzen ihrer Entscheidungen konkret vor Augen führen. Es ist offensichtlich, dass nicht nur die technisch-administrative, sondern auch die moralische Qualität der Allokation dringend benötigter Ressourcen die Höhe des erzielbaren Versorgungsnutzens festlegt. Die Good Governance des COVID-19-Pandemiemanagements ist keine theoretisch-abstrakte Überlegung. Es gilt, katastrophales, weil Menschenleben forderndes moralisches Versagen zu verhindern (Ghebreyesus, 2021).


Dr. sc. med. Dipl.-Kfm. Thomas Kapitza ist Affiliate des IBME und leitet das Med & Econ Ethics Lab IBME in Zürich.


Literatur

Badano G If You’re a Rawlsian, How Come You’re So Close to Utilitarianism and Intuitionism? A Critique of Daniels’s Accountability for Reasonableness Health care analysis: HCA: journal of health philosophy and policy 2018 26 1 1 16 10.1007/s10728-017-0343-9 28332130 PMC5816125
Berkley, S. (2020), COVAX explained, https://www.gavi.org/vaccineswork/covax-explained (22. Juli 2021).
Biller-Andorno, N. (2020), PubliCo Project, Universität Zürich, https://www.ibme.uzh.ch/en/Biomedical-Ethics/Research/Ongoing-Research/Public-Health-Ethics/PubliCo.html (22. Juli 2021).
Daniels, N. (2008), Just health: Meeting health needs fairly, Cambridge University Press.
Ghebreyesus, T. A. (2021), WHO Director-General’s opening remarks at 148th session of the Executive Board, WHO, https://www.who.int/director-general/speeches/detail/who-director-general-s-opening-remarks-at-148th-session-of-the-executive-board (22. Juli 2021).
Joebges S Biller-Andorno N Ethics guidelines on COVID-19 triage-an emerging international consensus Critical care 2020 24 1 201 10.1186/s13054-020-02927-1 32375855 PMC7202791
Kapitza, T., F.-H. Greiff und K. Mann (2020), Das Gesundheitssystem in der Post-COVID-19-Epoche — Die Pandemiekrise und neue Wege des Pandemiemanagements.
Rawls, J. (1999), A theory of justice (Rev. ed.), Belknap Press of Harvard University Press.
Spitale G., S. Merten, K. Jafflin, B. Schwind, A. Kaiser-Grolimund und N. Biller-Andorno (2020), [Protocol] PubliCo. A new risk and crisis communication platform to bridge the gap between policy makers and the public in the context of the COVID-19 crisis, Preprint, 30. November, https://zenodo.org/record/4551386#.YPnZ1G5CTLs.10.2196/33653 PMC8562419 34612823
